# Review of MR-Guided Radiotherapy for Esophageal Cancer

**DOI:** 10.3389/fonc.2021.628009

**Published:** 2021-03-22

**Authors:** Sangjune Laurence Lee, Michael Bassetti, Gert J. Meijer, Stella Mook

**Affiliations:** ^1^ Department of Oncology, Division of Radiation Oncology, Tom Baker Cancer Centre, University of Calgary, Calgary, AB, Canada; ^2^ Department of Human Oncology, University of Wisconsin Hospital and Clinics, Madison, WI, United States; ^3^ Department of Radiation Oncology, University Medical Center Utrecht, Utrecht University, Utrecht, Netherlands

**Keywords:** MRI, esophageal cancer, adaptive radiotherapy, respiratory motion, cardiac toxicity

## Abstract

In this review, we outline the potential benefits and the future role of MRI and MR-guided radiotherapy (MRgRT) in the management of esophageal cancer. Although not currently used in most clinical practice settings, MRI is a useful non-invasive imaging modality that provides excellent soft tissue contrast and the ability to visualize cancer physiology. Chemoradiation therapy with or without surgery is essential for the management of locally advanced esophageal cancer. MRI can help stage esophageal cancer, delineate the gross tumor volume (GTV), and assess the response to chemoradiotherapy. Integrated MRgRT systems can help overcome the challenge of esophageal motion due to respiratory motion by using real-time imaging and tumor tracking with respiratory gating. With daily on-table MRI, shifts in tumor position and tumor regression can be taken into account for online-adaptation. The combination of accurate GTV visualization, respiratory gating, and online adaptive planning, allows for tighter treatment volumes and improved sparing of the surrounding normal organs. This could lead to a reduction in radiotherapy induced cardiac toxicity, pneumonitis and post-operative complications. Tumor physiology as seen on diffusion weighted imaging or dynamic contrast enhancement can help individualize treatments based on the response to chemoradiotherapy. Patients with a complete response on MRI can be considered for organ preservation while patients with no response can be offered an earlier resection. In patients with a partial response to chemoradiotherapy, areas of residual cancer can be targeted for dose escalation. The tighter and more accurate targeting enabled with MRgRT may enable hypofractionated treatment schedules.

## Introduction

Esophageal cancer is the seventh most common type of cancer worldwide with the sixth most common cause of cancer-related death (1). Currently, neoadjuvant chemoradiotherapy (nCRT) followed by an esophagectomy is standard of care for patients with locally advanced resectable esophageal carcinoma (2, 3). Definitive chemoradiotherapy is the preferred approach for unresectable locally advanced esophageal cancer or for patients who decline or are unfit for surgery (4, 5). Thus, radiotherapy plays an important role in the treatment of esophageal cancer. Although nCRT results in an increase in R0 resection rate, locoregional control and improved overall survival, 5-year overall survival remains poor after trimodality treatment. Moreover, after definitive CRT, disease persistence and locoregional recurrence are common modes of treatment failure, especially in the primary tumor region (6, 7). These poor outcomes warrant improvements in radiotherapy for esophageal cancer patients. This article will provide an overview of the potential benefit and future role of MRI and MR-guided radiotherapy (MRgRT) in esophageal carcinoma.

## The Role of MRI in Esophageal Cancer

### Staging

Endoscopic ultrasound (EUS), computed tomography (CT), and positron emission tomography (PET) are typically used for initial staging of esophageal cancer (8). However, all these imaging techniques have limitations with regard to accurate staging, precise tumor delineation for radiotherapy and accurate response assessment after CRT. MRI is a non-invasive technique that provides excellent soft tissue contrast and allows for imaging of cancer physiology. Using T2-weighted (T2W) and diffusion-weighted imaging (DWI), stage T1 tumors can be detected in 33% of cases, T2 in 58%, T3 in 96% and T4 in 100%. MRI has a sensitivity of 38-62% and specificity of 68-85% for N-staging, making it a useful alternative especially in cases where the endoscope cannot pass an obstructing tumor (9). While MRI has had limited historical utilization in esophageal cancer, advances in MRI technology, including faster pulse sequences, cardiac and respiratory gating and surface coils, have improved the resolution of MRIs (10, 11). As these techniques continue to advance it promises greater use of MRI staging for esophageal cancer.

### Delineation

Accurate tumor delineation is essential to ensure adequate target coverage while limiting dose to surrounding organs at risk (OARs). Accurate gross tumor volume (GTV) delineation is especially important when cone down or boost strategies are applied. Delineation of the GTV of locally advanced esophageal carcinoma is usually based on CT, FDG-PET, endoscopy, and EUS. Despite this multimodality approach for tumor delineation, the interobserver variability remains substantial, especially in cranial caudal direction (12). The excellent soft tissue contrast of MRI could potentially increase the accuracy of GTV delineation. The GTV appears smaller on breath hold T2W and DWI compared to conventional PET-CT which is acquired during free-breathing. Moreover, the addition of DWI to T2w MRI reduced the variability of the caudal border in tumors involving the GE-junction, showing the potential value of DWI in these cases (12). In a study of 42 patients with esophageal squamous cell carcinoma who underwent breath hold CT and DWI MRI followed by an esophagectomy, the difference in tumor length between CT and pathology was 3.6 mm while the difference in length between DWI and pathology was as low as 0.5 mm (13). Despite the excellent soft tissue contrast provided by MRI a recent study showed that MRI based target delineation did not lead to reduced interobserver variability (12). This might be due to the limited observer experience to date with contouring esophageal tumors on MRI and image acquisition characteristics (axial plane only, slice thickness of 6.5mm).

### Response Assessment

After trimodality treatment, approximately one third of patients have a pathological complete response (pCR) (14). Patients who achieve a complete response after nCRT are likely to be unnecessarily exposed to the risks of esophagectomy, with up to 5% mortality, substantial morbidity and a substantial impact on quality of life (14, 15). Unfortunately, current techniques do not reliably identify complete responders (16). If these patients could be accurately identified prior to surgery, surgery might be omitted without jeopardizing outcomes.

Conversely, nearly one fifth of patients have more than 50% vital residual tumor cells in the tumor bed at histopathological examination after nCRT and are considered non-responders. These non-responders are exposed to nCRT related toxicity, probably without benefit. Therefore, accurate identification of non-responders early during the course of nCRT may allow for alternative treatment strategies, such as neoadjuvant treatment intensification, change in chemotherapy, or termination of ineffective neoadjuvant treatment and early surgery.

A meta-analysis of the current literature examining the diagnostic accuracy of clinically routine studies such as endoscopic biopsies, EUS, and PET-CT for detecting residual disease after nCRT showed that single modalities were insufficiently accurate (16). Another meta-analysis on the ability of various imaging modalities for detecting pathological complete response (pCR) showed pooled sensitivities of 0.35, 0.62, 0.01, and 0.80 and pooled specificities of 0.83, 0.73, 0.99, and 0.83 for CT, PET-CT, EUS and MRI respectively (17).

DWI and the derived apparent diffusion coefficient (ADC) and intravoxel incoherent motion (IVIM) models reflect tissue cellular density, extracellular-space tortuosity, and the integrity of cellular membranes (18). Recently, promising results for response prediction have been reported for this functional imaging modality. Baseline DWI prior to CRT therapy, interim DWI midway through treatment, and the change in between baseline and interim imaging have been found to be prognostic and predictive biomarkers (19–24). The relative change in ADC during the first 2 weeks of CRT appears to be the most predictive for residual cancer with a sensitivity of 100% and specificity of 75% (19, 20).

In addition to DWI, dynamic contrast enhanced (DCE) MRI, which involves the serial acquisition of T1-weighted images, before, during, and after the injection of a paramagnetic contrast agent such as gadolinium, provides further insight into the nature of tumor tissue and its close surroundings. DCE imaging reveals characteristics related to tumor vasculature permeability and extravascular extracellular volume (25). DCE imaging can be used to help identify esophageal carcinoma, lymphatic metastases and also predict response to CRT (26–28). Although the performance of DWI and DCE MRI as a single modality are promising, combinations of imaging modalities or MRI pulse sequences, may provide complementary value and could further improve the prediction of response to CRT (24, 26, 29).

Similarly, the preSANO trial showed that after nCRT, the use of biopsies, FNA, EUS, in combination with PET-CT could identify 70-90% of patients with more than 10% residual carcinoma in the esophagectomy specimen (30). More recently, the prospective PRIDE study aims at the development of a multimodal prediction model including MRI that not only predicts the patients’ individual probability of a pCR after nCRT, but also identifies non-responders and patients who are likely to develop distant metastases in the near future (31). Both the SANO and ESOSTRATE trials are comparing active surveillance with immediate surgery in esophageal cancer patients who have achieved a clinical complete response, predicted by PET-CT and endoscopic biopsies, after nCRT (32, 33).

## Rational for MR-Guided Radiotherapy in Esophageal Cancer

Integrated MRI-linear accelerator systems (MR-linacs) provide the ability to adapt the treatment based on daily changes in shape, size and position of the tumor and surrounding tissue in order to increase the accuracy of treatment delivery (19, 20). Due to the enhanced soft-tissue contrast, online MRI will allow real-time tumor visualization both before and during beam delivery. In combination with advanced online motion-compensation, MRgRT could well improve tumor targeting accuracy, allow for smaller planning target volume (PTV) margins and consequently result in a reduction of normal tissue exposure with a potential decrease in treatment related toxicity. Moreover, highly accurate tumor targeting with small PTV margins may enable hypofractionation and less toxic dose escalation to eradicative dose levels, potentially omitting the necessity of surgery to control the macroscopic tumor. Daily and even intrafraction plan adaptation and dose painting based on anatomical changes, tumor regression and functional MR imaging will further refine dose escalation and might provide an organ-sparing treatment strategy for a growing number of patients. The potential advantages of MRgRT for esophageal cancer will be discussed below.

### Online Interfraction Tumor Shape Adaptation

The primary tumor, involved nodes and the clinical target volume (CTV) consisting of the peri-esophageal fat often can hardly be discriminated on CBCT. This is particularly true for tumors located in the distal esophagus subject to respiratory and cardiac motion. This is the most common tumor location in the Western world, and often involves the proximal part of the stomach. Hence, set-up corrections are typically performed by online registration of the bony anatomy visible on CBCT, instead of direct matching on the tumor. The interfractional variation of the tumor position and shape in relation to the bony anatomy can be substantial and consequently large PTV margins are required to encompass esophageal tumor (34). Online high-quality MRI facilitates online tumor matching, reducing CTV to PTV margins. A recent study has demonstrated that a 10 mm PTV margin can provide CTV coverage in 89% of cases where daily set up position is based on a bone match (35). Only a modest improvement in CTV coverage to 93% could be achieved with a soft tissue, MRI-guided, CTV match with the same 10 mm margin. This reflects the considerable day-to-day CTV shape changes, especially for distal esophagus and gastroesophageal junction (GEJ), which regularly occurred over the course of treatment and could not be corrected by translational shifts based on soft-tissue registration. This partly explains the modest improvement of geometric coverage of the CTV with online MR-guided soft tissue matching and indicates that correction for the largest interfraction positional variation can only be achieved by daily online adaptation of the target and online replanning (35).

**Figure 1:**
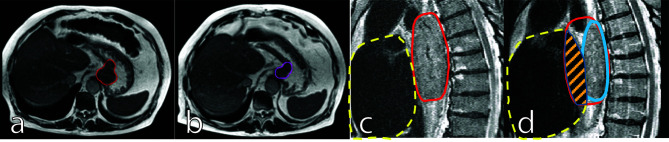
Mid treatment gastroesophageal adenocarcinoma tumor regression: Images are inhale breath hold 0.35T True Fast Imaging with Steady-State Free Precession (TRUFI) at baseline [**(A)**, red outline] and on fraction 10 of chemoradiation therapy [**(B)**, purple outline]. Sagittal views of thoracic squamous cell carcinoma tumor regression depicted 1.5T T2-weighted navigation triggered imaging at baseline [**(C)**, red outline] and on fraction 19 of chemoradiotherapy [**(D)**, blue outline]. In **(C, D)**, the dashed yellow shows the heart contour and the striped orange area shows regression from the overlap of the original tumor volume and the heart volume.

In addition to positional variation of GTV and CTV, substantial tumor volume regression during the course of nCRT can be visualized on MRI. By the fifth week of treatment, esophageal tumors can decrease by 28% of the initial volume (36). This tumor regression will predominantly result in deformation of the target and, as a consequence, OARs, especially the heart, could move into the initial GTV, thereby increasing the radiation dose to the heart and contributing to cardiac toxicity (**Figure 1**). The effect of tumor regression on the anatomical configuration can only be appreciated with online MR-guidance and corrected for by an online adaptive workflow where a new treatment plan is generated based on the anatomy of the day. This procedure, also referred to as adapt-to-shape (35) or stereotactic MR-guided adaptive radiation therapy (SMART) (37), will correct for interfraction variation.

### Dealing With Intrafraction Tumor Motion

Intrafraction motion due to respiratory motion revealed by cine MRI average 12–13 mm in the cranial-caudal (CC) direction, 2.5–5 mm in the anterior-posterior (AP) direction, and 2.7 mm in the left-right (LR) direction (38, 39). Lower esophageal tumors and GEJ tumors exhibit the largest motion and variability of motion during the respiratory cycle due to their proximity to the diaphragm (34, 40). In general, respiratory motion of esophageal tumors will cause a decrease in the sharpness of the dose gradient at the PTV edge, predominantly in CC direction, once the position of the target volume has been properly identified (41). Although the intrafraction motion of esophageal tumors can be categorized as modest and seldom leads to systematic errors, motion management techniques (e.g. respiratory gating, or mid-position techniques) are required to bring down CTV-to-PTV margins to 2–3 mm-levels in future treatments. Moreover, drift during treatment can be observed and although in general drifts are small with a mean of 1.5 mm, outliers up to 11 mm can occur (39).

MRgRT allows for online tumor motion monitoring, which affords the option to intervene in case of extreme anatomical changes and drifts are observed. Moreover, respiratory gating can mitigate the effect of respiratory motion and reduce the required PTV (42). On conventional linear accelerators, respiratory gating is performed using external surrogates, but the correlation between such surrogates and tumor motion can vary substantially (43). As such, image guidance is of utmost importance for accurate respiratory gating to avoid a geographical miss. MRI allows real-time position confirmation during gated treatment by tracking the GTV, ensuring accuracy of the treatment.

### Reducing Treatment-Related Toxicity

Smaller CTV to PTV margins will result in less dose to the surrounding organs at risk and thereby will theoretically decrease treatment related toxicity. In patients undergoing CRT for esophageal cancer, up to 10.8% develop symptomatic cardiac toxicity (44). Institutional retrospective and database analyses show that compared to patients who undergo esophagectomy alone, those who undergo nCRT have a significantly increased risk of grade 3 or higher cardiac events and that higher radiation doses to the heart correlates with a higher incidence of cardiac events (45–47). In a prospective phase II trial by Lin et al, 145 patients with esophageal cancer were randomized to definitive treatment with proton beam therapy or photon-based intensity-modulated radiation therapy. At a median follow up of 44 months, the total toxicity burden was lower in the proton beam therapy arm, with pronounced numeric differences in cases of atrial fibrillation, asymptomatic effusions, lower-grade pneumonitis, acute respiratory distress syndrome (ARDS) and reintubation. This study demonstrated that the dosimetric advantages of proton therapy resulted in lower rates of toxicity (48). Similar benefits could be expected from daily online adaptive MR-guided radiotherapy plans with tight CTV to PTV margins. MRL treatments using maximum inspiration breath hold under real time MRI tracking can help reduce treatment volumes. In a dosimetric analysis, compared to free breathing treatments on conventional CBCT guided radiotherapy, maximum inspiration breath hold MRL treatments for GEJ tumors can reduce the PTV from 1275 cc to 689 cc with a corresponding decrease in mean heart dose from 27.8 Gy to 20.9 Gy (42). While photon-based MRL treatments may have larger volumes of low dose coverage of OARs, due to uncertainties of the location of the Bragg peak, proton-based treatments are likely to have larger volumes of high dose coverage of proximal OARs. Future studies are warranted to compare the toxicity burdens between photon-based MRL treatments and proton therapy. The ability to visualize moving soft-tissue tumors with MRI and the dosimetric advantages of the Bragg peak with proton therapy could be combined in a hybrid system for MR-integrated proton therapy (MRiPT). Although MRiPT is still in its infancy, research is currently underway to develop prototype systems for clinical use (49). In addition to MR-guided daily plan adaptation and PTV margin reduction with consequently better sparing of OARs, MRI may also provide a way to detect subclinical cardiac toxicity after CRT by visualizing areas of myocardial fibrosis and changes in ejection fraction (50).

Besides limiting the radiation dose to the heart, smaller margins with MRgRT can also reduce the dose to the lungs and stomach. Grade 2 or higher radiation pneumonitis affects 5-7% of patients undergoing intensity-modulated radiotherapy for esophageal cancer, with greater incidence seen at higher lung V20 doses (51). Recent studies indicate that the ratio of the planning target volume to the total lung volume and the mean lung dose are important for predicting the probability of developing severe acute radiation pneumonitis (52). In patients who undergo esophagectomy, anastomotic leak rates range from 0–24% and are the cause of 90% of postoperative mortalities (53). The relationship between nCRT and rates of anastomotic leaks is controversial. The odds ratio for developing an anastomotic leak is 5.37 within the radiation field compared to anastomoses outside the radiation field (54, 55). However in studies comparing patients treated with neoadjuvant radiation therapy to resection alone, there was no difference in anastomotic leak rates (56, 57). Target definition at the GEJ is challenging and daily variation in this area can be substantial, therefore accurate dose accumulation in the area will be difficult, which might explain the conflicting results.

## Targeted Dose Escalation

Although progress has been made in the treatment of esophageal cancer, treatment still fails in most patients due to locoregional recurrences and the development of metastatic disease. The majority of local recurrences after definitive chemoradiation occurs within the GTV, suggesting a potential benefit of dose escalation in patients unfit for surgery or with unresectable disease (6, 7). Furthermore, the generally applied tumor radiation dose of 41.4 to 50.4 Gy is far below the commonly used doses at other primary tumor sites, such as lung and head and neck tumors. Patients treated with nCRT might benefit from dose escalation by increasing the chance of achieving a pCR. Moreover, patients with a pCR have a favorable prognosis (58) and it could be argued that surgery might be safely omitted in these patients.

Currently, results of dose escalation studies are inconsistent. A landmark randomized trial INT-0123 (RTOG 94-05) revealed that sequential dose escalation to 64.8 Gy did not translate into an increase in local control or overall survival in esophageal cancer. Radiotherapy techniques have evolved dramatically since the era of the INT-0123 trial and several retrospective and non-randomized prospective studies have shown an increase in local control after dose escalation (59–61). The ARTDECO trial, published in abstract form in 2020, randomized inoperable esophageal cancer patients (61% squamous cell carcinoma and 39% adenocarcinoma) to conventional CRT with a simultaneous integrated boost to a total of 61.6 Gy. Although modern radiation techniques were used, local progression free survival and overall survival were not statistically different between the two groups while the dose escalated arm had higher rates of grade 4-5 toxicity (62). The location and histology of the tumor may influence outcomes of dose escalation studies. Lower esophageal tumors are more challenging to treat. They tend to be adenocarcinoma which are more radioresistant than squamous cell carcinoma, have more cardiac and respiratory motion due to proximity to the heart and diaphragm, are pressed tightly to the adjacent heart, and are limited by proximity and radiosensitivity of the stomach.

The inconsistent results of dose escalation regarding local control and overall survival might also be due to the lack of patient selection. Careful selection of patients for a sequential boost based on the initial PET-CT response to standard CRT showed promising results (63).

## Future Prospects of MRI and MR-Guided Radiotherapy in Esophageal Cancer

Currently, at the UMC Utrecht the first patients with esophageal cancer are being treated on the 1.5T MR-Linac with reduced margins. Patients receive standard fractionated nCRT with reduced PTV margins (**Supplementary Materials**, **Figure S1**). This combined R-Ideal phase 1b-2a study with smaller PTV margins will serve as a proof of concept and the workflow and technology will be further optimized for future innovative treatments, such as dose escalation (64).

**Figure 2:**
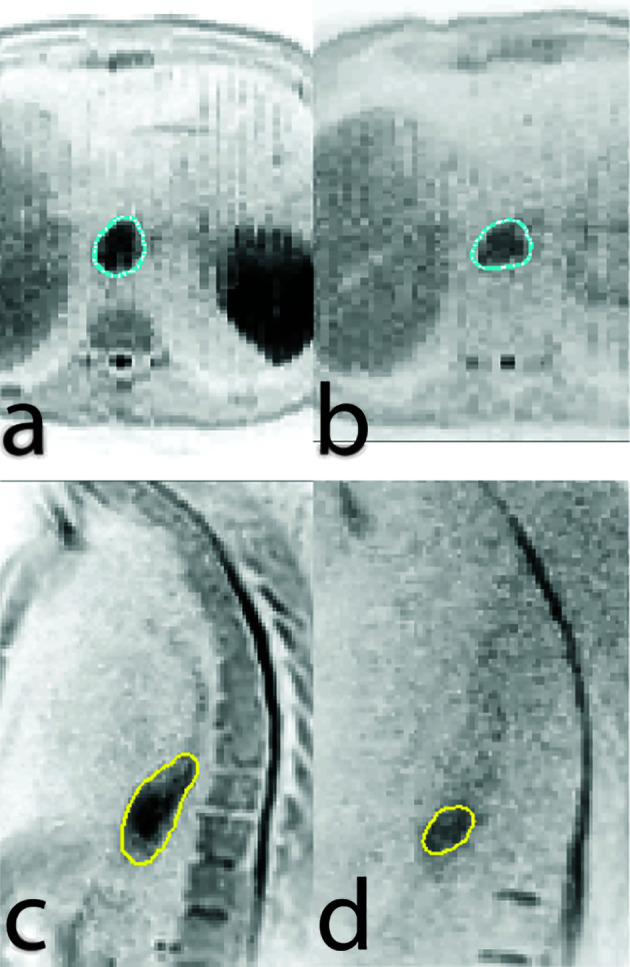
Axial **(A, B)** and sagittal **(C, D)** views of a diffusion weighted imaging scan conducted at baseline **(A, C)** and at week five of chemoradiation therapy **(B, D)** showing regression of tumor size but persistent diffusion restriction.

MR guided radiotherapy provides an exciting opportunity to improve and personalize esophageal cancer treatment by various means. First, MRI appears to be promising in treatment response assessment to guide patient-tailored treatment strategies, such as dose escalation or organ preservation. Second, online MR guided radiotherapy will result in high precision daily adaptive radiotherapy with reduced margins, thereby reducing toxicity and enabling safe targeted dose escalation. Finally, functional MR-guidance allows for dose painting strategies based on biological information about the tumor in order to increase its efficacy, such as dose escalation to only the parts of the GTV that exhibit persistent tumor activity at the end of standard CRT (**Figure 2**) (65). Randomized trials are needed to demonstrate the effectiveness of MR-guided radiotherapy compared to conventional CBCT guided radiotherapy.

In addition, the increased accuracy of online MR guided radiotherapy, due to daily adaptation of target delineation and online replanning in combination with beam on imaging, might pave the way for hypofractionated dose escalation in esophageal cancer. Hypofractionated radiotherapy has the advantage of a shorter overall treatment time and a higher biological effectiveness. Future studies need to elucidate whether hypofractionated radiotherapy will improve outcomes in esophageal cancer in terms of local control, will lead to adequate functional outcomes and is safe in terms of esophageal toxicity.

## Data Availability Statement

The original contributions presented in the study are included in the article/**Supplementary Material**. Further inquiries can be directed to the corresponding author.

## Author Contributions

SL, MB, and SM were responsible for the conception of this review. All authors contributed to the article and approved the submitted version.

## Conflict of Interest

MB reports non-financial support from Viewray Inc, outside the submitted work, and MERCK Clinical Trial Support, Astra Zeneca Clinical Trial Support, EMD Serono Clinical Trial Support. SL reports non-financial support from ViewRay, outside the submitted work. GM and SM are both members of the international MR-linac Consortium, which is financially supported by Elekta. The UMC Utrecht Department of Radiotherapy is financially supported by Elekta.
