# Supplementary material for: Review of MR-Guided Radiotherapy for Esophageal Cancer
Source: Front Oncol. 2021 Mar 22;11:628009. doi: 10.3389/fonc.2021.628009 (PMC8019940; doi:10.3389/fonc.2021.628009)
Supplement: Supplementary file 1 [file DataSheet_1.docx]

**Supplementary material:**


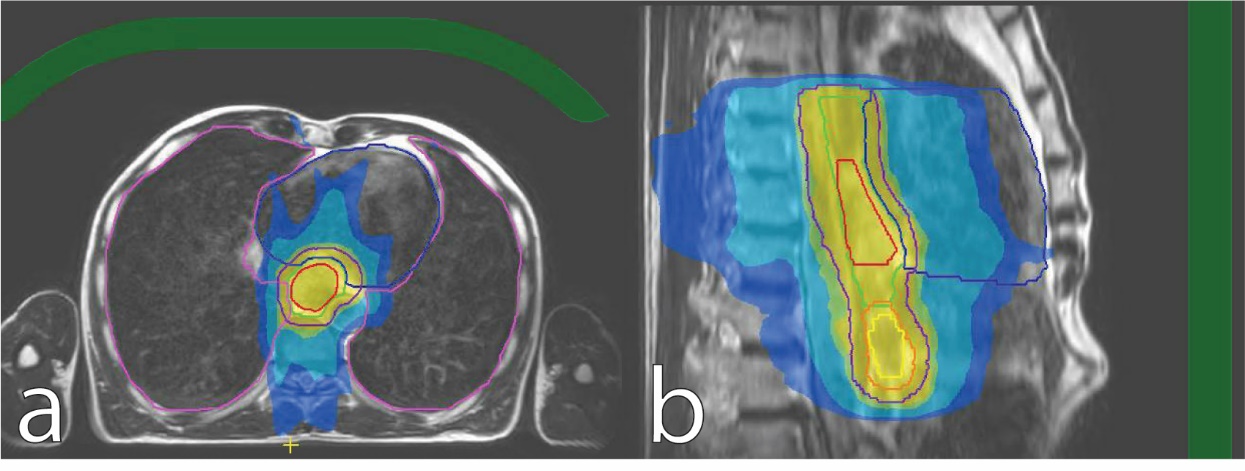


Figure S1: Axial (a) and transverse (b) views of 1.5T MR-Linac 3D T2-weighted online imaging with dose distribution of the 4th fraction of chemoradiation therapy. Red= GTVp, Yellow = GTVn, Green= CTVp, Orange = CTVn, Purple= PTVpn, Blue = Heart, Pink = Lungs. Isodoses: Yellow = 39,33Gy (95%), Light green = 37,26Gy (90%), Light blue = 26.91 Gy (65%), Dark blue = 20 Gy (48%).
